# Functional analysis of the F337C mutation in the *CLCN1* gene associated with dominant *myotonia congenita* reveals an alteration of the macroscopic conductance and voltage dependence

**DOI:** 10.1002/mgg3.1588

**Published:** 2021-01-28

**Authors:** Kevin Jehasse, Kathleen Jacquerie, Alice de Froidmont, Camille Lemoine, Thierry Grisar, Katrien Stouffs, Bernard Lakaye, Vincent Seutin

**Affiliations:** ^1^ Laboratory of Neurophysiology GIGA Institute Liege Belgium; ^2^ Department of Electrical Engineering and Computer Science Liège University Liège Belgium; ^3^ Laboratory of Molecular Regulation of Neurogenesis GIGA Institute Liège Belgium; ^4^ Neurogenetics Research Group Vrije Universiteit Brussel (VUB Universitair Ziekenhuis Brussel (UZ Brussel), Reproduction and Genetics Brussels Belgium

**Keywords:** channel gating, *CLCN1*, microscopy, myotonia, patch clamp

## Abstract

**Background:**

*Myotonia congenita* (MC) is a common channelopathy affecting skeletal muscle and which is due to pathogenic variants within the *CLCN1* gene. Various alterations in the function of the channel have been reported and we here illustrate a novel one.

**Methods:**

A patient presenting the symptoms of *myotonia congenita* was shown to bear a new heterozygous missense variant in exon 9 of the *CLCN1* gene (c.1010 T > G, p.(Phe337Cys)). Confocal imaging and patch clamp recordings of transiently transfected HEK293 cells were used to functionally analyze the effect of this variant on channel properties.

**Results:**

Confocal imaging showed that the F337C mutant incorporated as well as the WT channel into the plasma membrane. However, in patch clamp, we observed a smaller conductance for F337C at −80 mV. We also found a marked reduction of the fast gating component in the mutant channels, as well as an overall reduced voltage dependence.

**Conclusion:**

To our knowledge, this is the first report of a mixed alteration in the biophysical properties of hClC‐1 consisting of a reduced conductance at resting potential and an almost abolished voltage dependence.

## Graphical abstract

We describe a rare variant of the CLCN1 gene which was found in one family. Functional analysis demonstrated that the channel inserts normally into the membrane, but has a low conductance at −80 mV and a severely reduced voltage‐dependence.

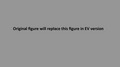

## 1 INTRODUCTION

*Myotonia congenita* (MC) is the most common genetic channelopathy affecting the skeletal muscle (Horga et al., 2013). Clinically, it is characterized by muscle stiffness following a voluntary movement, sometimes associated with a «warm up» phenomenon, which reflects the fact that this symptom improves with repetitive activity. By far the most frequent cause of MC is a pathogenic variant within the *CLCN1* gene (OMIM accession number 118425), which encodes the ClC‐1 channel protein (Jentsch & Pusch, 2018). This channel is the main contributor (~80%) of the resting conductance of skeletal muscle. It normally acts to dampen myocyte excitability after an action potential.

MC can be inherited in a dominant (Thomsen's disease) or recessive form (Becker's disease). Symptomatology is usually more severe in the latter because no rescue or attenuation is possible by normal channel proteins. Indeed, it is usually considered that severe myotonia is associated with a large reduction in the ClC‐1 conductance (by at least 80%), whereas smaller reductions are supposed to yield a mild phenotype (Colding‐Jørgensen, 2005).

ClC‐1 channels are homodimeric channels that present a double‐barreled structure (Ludewig et al., 1996; Middleton et al., 1996; Mindell et al., 2001; Park & Mackinnon, 2018). Extensive biophysical analyses have demonstrated the voltage dependence of these channels, which have two protopore gates (involving the individual subunits) and one common gate. This common gate affects both pores simultaneously across an intersubunit interface and is relatively slow, whereas the so‐called protopore gates are faster (Accardi & Pusch, 2000). Both gates need to be open to conduct Cl^−^ ions. The opening of both gates is voltage‐dependent, with an increase in open probability at potentials more depolarized than the muscle resting potential (−80 mV). However, the open probability is not 0 at −80 mV, but about 0.6 and 0.2, for the slow and fast gate, respectively (Accardi & Pusch, 2000). This explains why the channel both contributes to the resting conductance and is able to increase its opening probability during any depolarizing event (the time constant of the fast gate is fast enough for the ClC‐1 channel to contribute to the repolarization of the action potential).

More than 130 pathogenic variants in *CLCN1* have been identified, including small deletions, insertions, frame‐shifts, stop codons, missense, and splice‐site mutations (Imbrici et al., 2015). Generally, those variants in MC are responsible for a defect in the channel trafficking to the membrane (Vindas‐Smith et al., 2016) or a change in the biophysical properties by shifting the activation curve, reducing the unitary conductance, or changing the ion selectivity (Desaphy et al., 2013; Fahlke et al., 1997; Tsujino et al., 2011; Ulzi et al., 2012; Weinberger et al., 2012). It seems that, in dominant MC, a «hot spot» of mutations is observed in or close to exon 8, encompassing the regions between loops H and J of the channel (Fialho et al., 2007). In several cases, a major depolarizing shift of the curve describing the opening of the gate versus the voltage has been described, for example, in I290 M (Pusch et al., 1995) or F306L (Fialho et al., 2007). Such a shift will strongly reduce the ability of the channel to help repolarize the myocyte membrane at the end of the action potential. In other cases (e.g., A313 V), an almost complete abolition of the ClC‐1 current was found (Fialho et al., 2007).

Here, we report on a 23‐year‐old Belgian patient suffering from dominant MC associated with a heterozygous variant (F337C) in the same region. Since her infancy, she complained of transient muscle stiffness. She experiences difficulties mainly when initiating movements (such as when starting a walk, moving eyes, talking, or masticating). Her symptoms improve after repeated contractions, which is suggestive of a «warm up» phenomenon. They also tend to worsen with cold, for example, when washing her face with cold water. There is no complaint of muscle weakness after a rest.

The patient has non‐consanguineous parents. Her mother, as well as one grand uncle on the maternal side exhibit a similar semiology, suggesting a Thomsen type form of the disease. Her growth has been normal and she reports no other disease.

**FIGURE 1:**
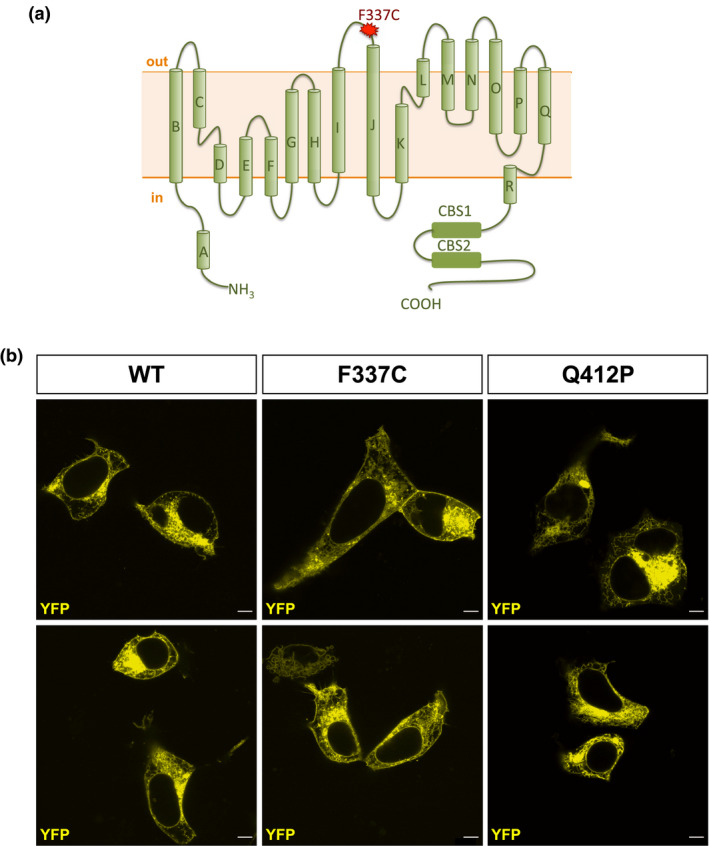
Subcellular localization of WT, F337C, and Q412P hClC‐1 channels. A, Schematic structure of a hClC‐1 channel subunit. The F337C mutation is located in the extracellular loop between I and J transmembrane domains. CBS is the cystathionine β‐synthase domain where ATP binds ClC‐1 and inhibits the common gating. B, Visualization of YFP‐tagged WT, F337C, or Q412P hClC‐1 channels in transfected HEK293 cells (scale bar: 5 µm). Note the clear membrane localization of WT and F337C channels, but not Q412P channels.

Medical examination shows general muscle hypertrophy, normal muscle strength, and attenuated myotatic reflexes. An electroneuromyogram has clearly confirmed the presence of myotonic burst discharges. Typical myotonic contractions can be clearly elicited by percussion of muscles of either the tongue or the thenar eminence. Serum creatine kinase was recently found to be at 220 IU/L (normal range: 15–130 IU/L). DNA isolated from blood lymphocytes was amplified by PCR and the 23 exons of *CLCN1*, as well as part of the flanking introns, were sequenced. The analysis showed that the patient is a heterozygous carrier of a c.1010 T > G, p.(Phe337Cys) substitution in exon 9 (Figure 1A). This alteration has already been observed (Derevenciuc et al., 2016), but no functional analysis has been performed. In silico analysis programs (SIFT°, PolyPhen2°, Mutation Taster°, and Align GVGD°) indicated that the substitution of this highly conserved amino acid is probably pathogenic, especially in view of a change from a hydrophobic amino acid to a cysteine. We therefore decided to investigate the trafficking and functionality of this mutant channel.

We first looked whether the F337C mutation affects the expression of the channel at the cell membrane. To appreciate the membrane location of F337C, we compared it with the one of WT and Q412P hClC‐1 channels. The latter were chosen as a control because they had previously been demonstrated to have a very small surface expression (Vindas‐Smith et al., 2016). We used the plasmid pRcCMV‐YFP‐hClC‐1 for transfection (Ronstedt et al., 2015). At first glance, it is clear that F337C hClC‐1 channels are expressed at the plasma membrane (and more so than Q412P), although more experiments are needed to quantify the density of F337C at the cell membrane.

**FIGURE 2:**
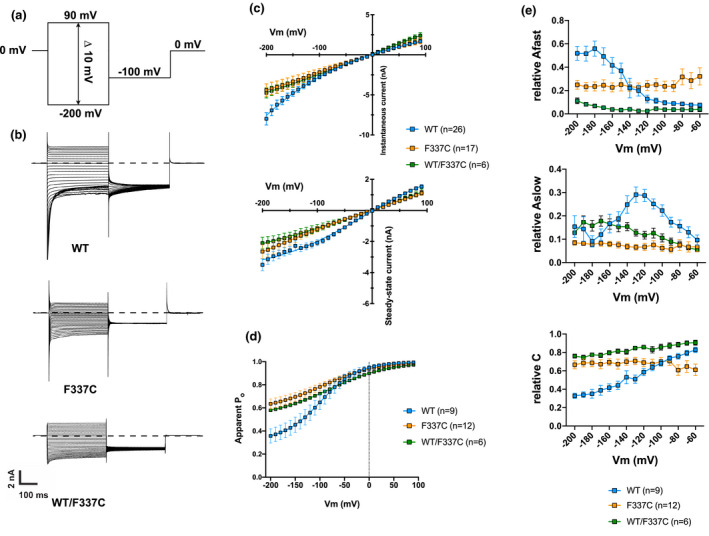
Functional analysis of WT and F337C hClC‐1 channels. A, Protocol used in all our experiments. B, Examples of current traces of WT, F337C, and WT/F337C hClC‐1. C, Current‐voltage curves of the instantaneous (top) and steady‐state (bottom) hClC‐1 currents. Instantaneous WT currents display an inward rectification, whereas F337C and WT/F337C currents are linear. Steady‐state F337C and WT/F337C currents are more linear than WT currents. D, Apparent P_o_‐voltage curve. The apparent P_o_ was obtained by measuring the tail current at −100 mV (post‐pulse) as a function of the voltage reached during the previous step (see supporting information). The P_o_ of F337C and WT/F337C is much less voltage‐dependent than that of WT. *E*. Kinetics of deactivating currents for WT, F337C, and WT/F337C hClC‐1 channels. See Eq.1 in the Methods for the fit. Each point is represented as a mean ± SEM of 9, 12, and 6 fits for WT, F337C, and WT/F337C currents, respectively. Note the reduction of the contribution of Afast and Aslow caused by the F337C mutation.

To evaluate the functional properties of the WT and F337C hClC‐1 channels, we used a classical voltage clamp protocol (see Figure 2A). In this protocol, the cells were held at 0 mV to strongly activate the fast and slow gates in symmetric chloride conditions (see SI), meaning that concentrations of intracellular and extracellular chloride were equivalent. Deactivation of the channels at negative potentials could be visualized as a biexponential decrease of the current. The instantaneous and the steady‐state currents were measured respectively at the beginning (~1 ms) and the end (~350 ms) of each pulse of voltage to obtain the IV curves shown in Figure 2C. In the WT, we observed a clear deactivation of the channel in the range of −200 mV to −100 mV. In F337C, the instantaneous current was much smaller in the same voltage range. When plotting the IV curves of the instantaneous currents from the WT and F337C, we observed in the mutant a loss of the typical inward rectification (Figure 2C) of these channels. As far as the steady‐state currents were concerned, F337C generated a linear current, smaller in amplitude than the one generated by the WT. In addition, the current of the variant was extremely linear, resembling a leak channel. In order to verify that this «leak‐type» current was indeed generated by the mutant, we performed the same recordings on non‐transfected cells (data not shown). No detectable current was measured. The conductance of the mutant around −80 mV was significantly smaller than the one of the WT channel, 11.88 ± 0.4 nS (n = 17) versus 21.99 ± 0.4 (n = 26) (ANOVA‐1 followed by Tukey's test, *p* < 0.001), but only by about 50%. The voltage dependence of the channel activation was obtained from the apparent P_o_ as a function of the membrane voltage (Figure 2D). F337C decreased the voltage dependence of the apparent P_o_ without affecting its V_1/2_ (see Table S1 for the quantification).

To quantify the loss of current deactivation in F337C, we fitted the curves using a double‐exponential function as described in the methods, including the residual current, between −200 and −60 mV. There was a significant difference between WT and F337C in the fast and slow amplitude components, as well as in the residual component (Figure 2E). Thus, the progressive increase in the weight of the fast deactivation (A fast) at more negative potentials was lost in the mutant, as was the weight of the slow deactivation in the −100 to −150 mV range. In addition, the relative weight of the constant current was high at all voltages in the mutant, whereas it decreased toward the more negative potentials in WT channels, because of the preponderance of current deactivation. The apparent lack of deactivating current in F337C is consistent with a higher apparent P_o_ at hyperpolarized potentials.

Given the dominant nature of the F337C variant, we transfected HEK293 cells with equal amounts of WT and F337C cDNA to generate heteromeric channels, similarly to what happens in heterozygous patients. We performed the same current analysis as described above. WT/F337C heteromeric channels (n = 6) displayed properties similar to those of F337C homomeric channels, with a reduced conductance at −80 mV (11.35 ± 2.5 nS for WT/F337C vs. WT, ANOVA‐1 followed by Tukey's test, *p* < 0.05) (Figure 2, Table S1). Therefore, we can assume that the total ClC‐1 current is mainly generated by F337C rather than WT in heterozygous patients carrying this variant.

These data show that the F337C mutation significantly decreases the macroscopic conductance at the resting membrane potential (about 50% of the conductance of WT hClC‐1 channels), and strongly reduces the channel's voltage dependence. Functionally, this means that the channel is able to sustain the resting membrane potential of this patient's myocytes. However, it will not be able to massively activate during action potentials and to powerfully bring the membrane potential back to its resting value. Therefore, the likelihood of repetitive action potentials underlying myotonic burst discharges will be much increased. The clear myotonic phenotype of the patient bearing this mutation suggests that the repolarizing effect of hClC‐1 during an action potential may be more important than its resting activity to protect normal fibers from abnormal repetitive discharges, a question that is somewhat controversial (Pedersen et al., 2016).

To our knowledge, the phenotype of the F337C mutant is rather original in the sense that most pathogenic variants either almost completely abolish channel conductance (which was not the case here) or shift voltage dependence to more depolarized potentials (whereas V_1/2_ was not changed, but voltage dependence was reduced in our case) (Imbrici et al., 2015). The question arises whether this phenotype may be due to the cysteine residue of the mutant channel. It is possible that this replacement will yield an abnormal intramonomer or intermonomer disulfide bridge which would be responsible for this specific channel phenotype and also affect the WT subunits in heteromeric configurations, just like the neighboring variant T335N affects WT subunit functionality by shifting the open probability to more depolarized potentials (Imbrici et al., 2016). Further experiments using reducing conditions, alternative substitutions of the phenylalanine, membrane expression, and measuring the P_o_ of the fast and slow gating separately will help answer this question.

In summary, we have characterized a mutant of the hClC‐1 channel protein, F337C, to correlate it with the myotonic symptomatology of this patient. When expressed in HEK293 cells, this mutant seems to insert normally in the membrane, but has major functional alterations consisting of a reduced steady‐state conductance and a loss of voltage dependence.

## CONFLICT OF INTERESTS

The authors declare no conflicts of interest with respect to the research, authorship, or publication of this article.

## AUTHORS’ CONTRIBUTION

KJe, AdF, and CL performed the transfections and the electrophysiological experiments. KJa implemented the curve fitting. KS genotyped the patient. TG was in charge of the patient's treatment and had the idea of the project. BL did the site‐directed mutagenesis. VS supervised the whole project. KJe and VS wrote the manuscript.

## Supporting information

Supplementary Material

## Data Availability

The data that support the findings of this study are available from the corresponding author upon reasonable request.
