# Supplementary material for: Functional analysis of the F337C mutation in the CLCN1 gene associated with dominant myotonia congenita reveals an alteration of the macroscopic conductance and voltage dependence
Source: Mol Genet Genomic Med. 2021 Jan 28;9(2):e1588. doi: 10.1002/mgg3.1588 (PMC8077071; doi:10.1002/mgg3.1588)
Supplement: Supplementary file 1 — Supplementary Material [file MGG3-9-e1588-s001.docx]

**SUPPLEMENTARY INFORMATIONS**

METHODS

Informed consent of the patient

The Ethics Committee of the local institution deemed that this research did not have to be submitted to it, provided that a written agreement was obtained from the patient. This agreement was obtained by TG.

Gene sequencing

Sanger sequencing of the gene (NCBI ref sequence: NM_000083.3) was performed for all coding exons and part of the flanking introns using standard procedures. This variant has been submitted to ClinVar (SUB7196737).

Mutagenesis

Plasmid pRcCMV-YFP-hClC-1 was a generous gift of Dr. C. Fahlke. Mutations were introduced into the plasmid by replacing a 1.1 kb SpeI/AfeI fragment by a mutated (either F337C or Q412P) double stranded gBlock DNA Fragment (IDT-DNA, Belgium). The sequences were verified by sequencing (GIGA-Genomics platform, Liège University).

Electrophysiology

Experiments were performed on transiently transfected HEK-293 cells. The cells were transfected with 1 µg plasmid pRcCMV/YFP/hClC-1, containing the full-length WT hClC-1 cDNA and/or the F337C hClC-1, using TransIT®-LT1 (Mirus Bio, Madison, WI). Cells were used 24 h after transfection. Whole-cell patch-clamp experiments were performed using classical methods. Data was acquired at room temperature (21-24°C) using an EPC-10 amplifier (Heka, Lambrecht, Germany) and Patchmaster software (Heka, Lambrecht, Germany). Low-resistance pipettes (≈ 3 MΩ) were pulled from filamented borosilicate glass tubing (2.0 mm outer diameter, 0.42 mm wall thickness; Hilgenberg) with a P87 puller (Sutter Instruments, Novato, CA, USA). Recordings in which the series resistance was ≥ 10 MΩ were discarded. The composition of the extracellular solution which superfused (@ 2 ml/min) the cells was (in mM): 140 NaCl, 4 KCl, 2 CaCl_2_, 1 MgCl_2_ and 5 HEPES, with the pH adjusted to 7.4 using NaOH. The composition of the internal solution, was (in mM): 140 NaCl, 2 MgCl_2_, 5 EGTA, 10 HEPES, pH adjusted to 7.4 with NaOH. In this condition, the reversal potential of Cl^-^ was -1 mV. Cells were therefore held at 0 mV.

Data analysis

MatLab° was used to analyze the time course of current deactivation. An algorithm was developed to fit the decay of currents elicited between -200 and -60 mV with a sum of two exponentials and a time-independent component. The following equation was used :

Eq.1 I(t) = Afast . exp(-t/τfast) + Aslow . exp(-t/τslow) + C

where τfast and τslow are the time constants of the fast and slow components of current relaxation/deactivation, and Afast, Aslow, are the weights of the fast and slow components, and C is the steady-state component of the current. Relative Afast, Aslow, and C were defined as the ratio of the parameter on the sum of Afast, Aslow, and C.

The voltage dependence of channel activation was examined by plotting the apparent open probability (P_o_) as a function of the pre-pulse voltage ranging from -200mV to 90mV. The following formula was used for the fitting,

Eq.2 P_o_(V) = Min + (1-Min)/[1+exp((V_1/2_-V)/S]

where Min is the minimal value of P_o_, V1/2 is the half-maximal activation potential, and S is the slope factor. Both types of analysis were done on a subset of the recordings with the lowest noise in order to enhance their reliability.

Confocal imaging

HEK293 cells were plated in 24-well plates at a density of 1.8 10^5^ cells/well in DMEM (Lonza). They were transfected with either an empty plasmid or a plasmid containing cDNA of WT, F337C or Q412P channels. The latter were chosen as a control because they had previously been demonstrated to have a very small surface expression (Vindas-Smith et al., 2016). The concentration of all plasmids was 0.1 µg/µl. Cells were transfected using Xtreme Gene (Sigma Aldrich), diluted at 1/33 in Mix OptiMEM and applied at room temperature for 15 minutes. On the next day, « Cellview » dishes (Greiner Bio One) were coated with 100 µg/ml Poly-D-Lysine (Lonza) and left for 1h at 37°C. After rincing and trypsin (Gibco – ThermoFisher Scientific) digestion, the cells were plated (2 ml/dish). On the next day, cells were observed using an inverted Zeiss confocal microscope (LSM 880 Elyra S1).

Statistical analysis

Results are expressed as mean ± SEM. Statistical analysis was performed using Prism version 8 (Graphpad Software), using the adequate statistical test. P<0.05 was considered as significant.

| Variant | N |  | Min |  | V_1/2_ (mV) |  | S |
| --- | --- | --- | --- | --- | --- | --- | --- |
| WT | 9 |  | 0.29 ± 0.06 |  | -106.1 ± 12.3 |  | 36.7 ± 3.9 |
| F337C  WT/F337C | 12  6 |  | 0.56 ± 0.06**  0.56 ± 0.02* |  | -103.7 ± 10.9  -84.2 ± 8.7 |  | 53.9 ± 2.7**  60.9 ± 2.5** |

**Supplementary Table 1. Boltzmann parameters of activation curves of WT, F337C and WT/F337C hClC-1 channels.** The activation curve found in each experiment was fitted with a Boltzmann equation (Eq.2). The parameters are reported as mean ± SEM. N is the sample size, Min is the minimal (relative) open probability, V_1/2_ is the voltage at which activation is half-maximal and S is the slope factor. * = p < 0.05, ** = p < 0.01, ANOVA-1 followed by *post-hoc* Tukey’s tests, in which values of F337C and WT/F337C channels were compared to those of WT channels. The activation curves are shown in Fig. 2D.
